# Phthisis Pulmonalis, or Tubercular Phthisis

**Published:** 1882-10

**Authors:** 


### BOOK NOTICES, REVIEWS, ETC.

Phthisis Pulmonalis, or Tubercular Phthisis. By Gershom N. Brigham, M. D., Grand Rapids, Mich. Bœricke & Tafel: New York and Philadelphia. pp. 244. Price, $2.00. 1882.

The first thing to be said of this work is to commend it for its candor and moderation of expression. The next thing is to commend its author for his evident desire to find the grain of truth in the multiplicity of errors and contradictions by most writers upon the subject, and to present that truth without apparent reference to policy, professional pride, or other motive than for truth's own sake.

In treatment and hygiene he has certainly secured and given us much that is true; and from future experience he will, no doubt, be able to give us much more that is equally good in that direction.

On ætiology and pathology, however, he has not been so fortunate (though his efforts have here manifestly been equally well-intentioned); and upon this branch of the subject it appears to us something earnest should be said.

When will the profession in this country emancipate itself from its dependence upon the errors and contradictions in medical matters annually sent us from Europe; and when will our school abandon its reliance upon allopathic pathological fallacies, observe and think for itself, and establish a system of ætiology and pathology of its own that shall be true to nature, and as much in advance of that of the old school as our therapeutics are in advance of theirs?

Scarcely any two of the prominent writers throughout the world agree as to the nature or process of formation of tubercles; but, what is still worse, some of them even contradict themselves. This shows clearly enough that there must be something radically wrong in their way of looking at or of handling the subject, and Dr. Brigham must have found it anything but pleasant or satisfactory trying to extract truth from such a source. His greatest error, therefore, (and error it must be considered in trying to harmonize contradictions that nature does not tolerate), was in following such leaders and summarizing their conflicting theories, then leaving the reader to judge for himself as best he might.

For evidence that everything is contradictory in that field, we need only refer to the Doctor's own extracts from, and explanation of, different writers' theories (which an evident spirit of fairness to those authors and to his readers led him to give); but for still stronger proof of the fact, we cite the following: Virchow says of tubercular corpuscles, page 518, *Cellular Pathology:* "They are not by any means the first bungling products, unfortunate essays of organization, but were once well-grown elements," etc., while only four pages further on, viz., on page 522, when speaking of the large cells of cancer and contrasting tubercular corpuscles with them, he says: "Tubercle, on the contrary, is always a pitiful production, a new formation, from its very outset miserable."

Dr. Brigham gives the views "adopted in *Niemeyer's Theory and Practice,*"

which "regards tubercle as a new growth and as the outcome of cell-proliferation;" and "'standing in closest relation to connective tissue formations.'" And yet elsewhere Niemeyer, in speaking of the coagula left in the lungs after hæmoptysis, and when trying to enforce the doctrine that inflammation is the cause of tubercle, said: "It is precisely the rule that the originally dark brown nodule, by a lengthened process, becomes yellow and cheesy."

Nature has no such contradictions and tolerates none, and we may be sure that men are going wrong when they thus interpret her work. Virchow was trying to prove that tubercular corpuscles were from a proliferation of connective tissue-cells, when using the language first quoted from him, but, knowing nothing definite as to what they were and only *guessing* at it, he forgot himself and thus flatly contradicted his own assertion in the short space of four pages. Niemeyer's contradiction is about as bad, while all prominent foreign writers upon tubercle contradict each other more or less fully; and yet we, in this country, swallow their teachings and try to digest or harmonize them, because we think we must to appear learned, or to be popular, or for some other equally absurd reason, instead of observing and thinking for ourselves.

Dr. Brigham, however, must not think these strictures are alone for him. We are all about equally guilty in this respect; that is, the profession in this country demands, or, at least, expects, a re-hash of such absurdities from our writers, and so long as it does the dish will be served. When it asks for something better it will, no doubt, be forthcoming.

Instead of deceiving ourselves any longer with such borrowed trash, why don't our school take the much-despised psoric doctrine of Hahnemann and make it the foundation-stone upon which to build a monument to the simple truth in the ætiology and pathology of tuberculosis, and many other chronic diseases, that shall be grander than any temple ancient history mentions, or than Oriental fable ever conceived, and that shall equal anything that science has ever given us in any of her other departments?

To illustrate: Take that same despised psoric doctrine, which is as true in principle as any principle through which God governs life or the universe, and add to or build upon it the following *facts*, viz.: That, when eczema, tinea capitis, psoriasis, acne, impetigo, lepra, or other chronic skin diseases bearing more common names, as "salt rheum," "barber's itch," etc., etc., are suppressed, that is, "cured," as it is falsely called, by local treatment, they are simply translated internally, and *always*, sooner or later, seat upon some one or more of the mucous membrane: That when any one of them does become fixed and active, there, it excites an irritation that causes a greatly-increased flow of mucus and waste of albumen from the blood through that membrane: That such loss of albumen robs the muscular system of just so much of its only food, thus causing the great emaciation so characteristic of consumption, and, at the same time, throws all the constituents of the blood into a disproportion, leaving a relative excess of water, blood-corpuscle, fibrin, fatty-matters, salts, etc., in the circulation, which is unnatural and must cause serious disturbances: That the excess of water thus left causes the diuresis, night-sweats and dropsies of consumptives: That the blood corpuscles left in excess thereby lead to the primary hemorrhages of such subjects, and that later other of such corpuscles are decolorized by circulating in the too watery serum of their blood, are then congested in the capillaries of the lungs or other parts, where they shrivel into tubercular corpuscles, have some of the excess of fibrin extravasated and organized around them, when the whole becomes a tubercle: That the fibrin left in excess is in part extravasated to form the bands, cords and plates of fibrinous adhesions about the lungs that are universal in consumption, in part to inclose all tubercles, in part as fibrinous casts of bronchial tubes, and also, in part, into the lungs, "developing fibrinous masses, which [Dr. Brigham says] are the best of soil for tubercles:" That the excess of fatty-matters causes the fatty livers and other fatty degenerations or deposits of phthisis or other scrofulous diseases: That the excess of salts causes the enlarged joints of scrofulous subjects, ossifications, chalky deposits, calcareous concretions in the lungs, or like casts of the tubes or air-cells, etc., etc.

We might go even further and show cancer to be caused in some psoric constitutions, in a similar manner—encephaloma by an excess of the brain nourishing material being left in the blood by loss of albumen, and the abnormal growth or development in consequence, of brain cells in the eye, face, etc., where they do not belong, and produce the worst forms of cancer; epithelioma as the result of the food for epithelial cells being left in excess, and such cells being grown, in consequence, where they do not belong, and thus causing epithelial cancer; or we might show that the suppression of both gonorrhea and syphilis are very liable to cause consumption by their being driven internally, in many cases, to seat upon some mucous membrane, and cause a similar waste of albumen with all the other results above pointed out; or, yet again, we might show the *mechanical* production of consumption in stone-cutters, edge-tool and needle-grinders, coal workers, millers, cotton and woolen operatives, etc. (of whom Dr. Brigham speaks quite fully), by the dust they inhale exciting more or less constant irritation of the mucous membrane of the throat and bronchi and waste of albumen thereby, until that in itself may result in tubercles; or, what is more common, such irritation arouses some latent psoric, syphilitic or gonorrheal taint, and sets that to work in the way and with the results above shown; but both time and space forbid our entering upon those themes now.

What a monument have we here, then, to Hahnemann's doctrine of chronic diseases and to nature's extreme simplicity in the midst of such apparently inextricable complication and confusion; and what an unfolding of truth in the field of ætiology and pathology to contrast with and offset the errors and contradictions we have hitherto so greedily taken from the old school. Will our school, or our writers, longer hesitate from which to choose?

As to indications to guide us in the selection of remedies, Dr. Brigham has given us many that are valuable; but we miss some that we have learned by experience to most rely upon.

From Arsenicum we miss catarrhal aggravations from five to six P. M.; also aggravations of cough, etc., after midnight. But, at the same time, we can strongly indorse him in the following: Cough brought on or greatly aggravated in the evening on lying down, and in the morning on rising, both occurring every night and morning; great aggravation of cough, or great dyspnœa, or both, on lying down day or night—has to be bolstered up to a half or full sitting posture; rapid emaciation, although eating well or fairly; very acute or stitching pains in apex of right lung.

Under Belladonna there is an absence of some of its most prominent indications, in the beginning of chronic lung diseases, namely, a *hollow* cough, more hollow and different from that of croup; also a barking and harsh cough, something like croup; cough arising a little before or precisely at midnight (Arg. n.); violent stitching pains from the right side of the abdomen upward through the right lung to mamma, point of right shoulder, and inner border of right scapula; also chronic nasal or bronchial fluent catarrh, attended with rattling respiration in nervous or mentally active subjects.

In the indications for Calcarea carb. the Doctor has not given that prominence to the rapid growing of children or youth which its importance demands, and which we have seen confirmed in a great many cases. Instead of this he speaks under Phosphorus of the "petite figure" of Calcarea subjects, to contrast them with the more powerfully built frames of those in whom Phos. is often indicated. But in our experience Calc. is much the more frequently called for in the lung diseases of tall, slender and rapidly growing youth than is Phos. So marked, indeed, has this experience been that we have often taken the fact of our patient having grown rapidly in youth, and possessing a large frame, as the guide to the true remedy for that person in middle life or later, and found it to be Calcarea much more commonly than Phosphorus.

Crocus we miss entirely from the Doctor's list, with its asthmatic or wheezy cough, attended by frothy expectoration containing threads of translucent, whitish, or yellowish mucus, of the size of coarse spool cotton or fine twine; and aggravation of many or all its symptoms (especially if such threads are expectorated) by hot weather, a warm room, lying down, etc.

One of the greatest of all the characteristics of Mercurius in lung diseases is also wanting in Dr. B.'s work, namely: great aggravation from, or utter impossibility of, lying upon the *right* side. A knowledge of this one symptom of Merc. has enabled us to cure with it more cases of very serious lung diseases than through all its other indications combined. There is also an absence of another very characteristic symptom of this drug, and that is, *fugitive pains*, now here, then there, or anywhere, and changing place every hour, few hours, or day or two. On this symptom we have also cured many serious cases of acute and chronic diseases with Merc.—five times as many, at least, as from prescribing Pulsatilla on the same symptom.

But here we will tell all: they will seldom or never cure severe cases having either of these symptoms, if the patients are victims to that terrible curse, namely, *mercurial dentistry;* that is, wearing red rubber dental plates, or having amalgam fillings in teeth, unless they first have said plates or fillings removed. Of all the minor evils to mankind, we do not hesitate to say from much experience that this mercurial dentistry is one of the greatest.

Nux vomica, the Doctor has entirely omitted from his book, and this we are surprised at. No remedy surpasses and few equal it for *violent racking* paroxysms of cough, whether acute or chronic, and with or without expectoration; and aggravation of cough *after eating* and in the morning or forenoon. Also, when cough is attended by severe headache, pain or bruised sensation in stomach, hypochondria or bowels, and a dull or sub-acute soreness of the abdomen under pressure.

From Phosphorus we miss one of its greatest characteristics, viz., very fetid stool and flatus, the odor being exactly like lime that has been used at gas-works to desulphurize the gas. For fifteen years or more we have never failed to greatly relieve suffering, even in incurable chronic lung diseases, with Phosphorus, when this symptom was present, and have never failed to speedily cure any case of acute diarrhœa having this symptom, with a single dose of Phosphorus in high potency.

Another marked symptom of Phosphorus that is omitted from the book is acute pains in the chest, especially right side, which are greatly aggravated by even light pressure in the inter-costal spaces, and in that case aggravation from lying on the right side; whereas, aggravation from lying upon the left side is, in the generality of lung diseases, one of the greatest characteristics of Phosphorus.

Under Sepia the Doctor has omitted one of its most prominent indications, namely, excessively fetid sputa.

There are other remedies and symptoms to which we might give attention, did time permit; but as every physician has experience that differs more or less from that of others, each can supply for himself what he sees to be wanting. Dr. Brigham has given his experience in a very satisfactory manner, and if all would do as well we would soon have the homœopathic therapeutics of tuberculosis placed upon a much higher plane than they occupy at present.

Upon the subject of potencies and the repetition of doses we are glad to see that the Doctor has taken such advanced ground. It is as important to avoid the too frequent repetition of doses as to select the right remedy. How many cases do we remember in our earlier experience where the right remedy was given with the most highly satisfactory results for a short time, but giving it too often soon brought up great aggravations that could not be controlled, and which the patient never rallied from. And how much of this experience it required before we could say, and act up to it, "hands off," until the improvement flags very materially before giving another dose, or changing the remedy.

